# RNF20 and RNF40 regulate vitamin D receptor-dependent signaling in inflammatory bowel disease

**DOI:** 10.1038/s41418-021-00808-w

**Published:** 2021-06-04

**Authors:** Robyn Laura Kosinsky, Maria Zerche, Ana Patricia Kutschat, Asha Nair, Zhenqing Ye, Dominik Saul, Maximilian von Heesen, Jessica J. Friton, Ana Carolina Schwarzer, Nadia Paglilla, Shehzad Z. Sheikh, Florian Wegwitz, Zhifu Sun, Michael Ghadimi, Rodney D. Newberry, R. Balfour Sartor, William A. Faubion, Steven A. Johnsen

**Affiliations:** 1 Division of Gastroenterology and Hepatology, Mayo Clinic, Rochester, MN USA; 2 Department of General, Visceral and Pediatric Surgery, University Medical Center Goettingen, Goettingen, Germany; 3 Division of Biomedical Statistics and Informatics, Mayo Clinic, Rochester, MN USA; 4 Kogod Center on Aging and Division of Endocrinology, Mayo Clinic, Rochester, MN USA; 5 Center for Gastrointestinal Biology and Disease, University of North Carolina at Chapel Hill, Chapel Hill, NC USA; 6 Division of Gastroenterology, Washington University School of Medicine, St. Louis, MO USA; 7 Gene Regulatory Mechanisms and Molecular Epigenetics Laboratory, Division of Gastroenterology and Hepatology, Mayo Clinic, Rochester, MN USA

**Keywords:** Histone post-translational modifications, Gastrointestinal diseases, Experimental models of disease, Chronic inflammation

## Abstract

Despite the identification of several genetic factors linked to increased susceptibility to inflammatory bowel disease (IBD), underlying molecular mechanisms remain to be elucidated in detail. The ubiquitin ligases RNF20 and RNF40 mediate the monoubiquitination of histone H2B at lysine 120 (H2Bub1) and were shown to play context-dependent roles in the development of inflammation. Here, we aimed to examine the function of the RNF20/RNF40/H2Bub1 axis in intestinal inflammation in IBD patients and mouse models. For this purpose, intestinal sections from IBD patients were immunohistochemically stained for H2Bub1. *Rnf20* or *Rnf40* were conditionally deleted in the mouse intestine and mice were monitored for inflammation-associated symptoms. Using mRNA-seq and chromatin immunoprecipitation (ChIP)-seq, we analyzed underlying molecular pathways in primary intestinal epithelial cells (IECs) isolated from these animals and confirmed these findings in IBD resection specimens using ChIP-seq.The majority (80%) of IBD patients displayed a loss of H2Bub1 levels in inflamed areas and the intestine-specific deletion of *Rnf20* or *Rnf40* resulted in spontaneous colorectal inflammation in mice. Consistently, deletion of *Rnf20* or *Rnf40* promoted IBD-associated gene expression programs, including deregulation of various IBD risk genes in these animals. Further analysis of murine IECs revealed that H3K4me3 occupancy and transcription of the Vitamin D Receptor (*Vdr*) gene and VDR target genes is RNF20/40-dependent. Finally, these effects were confirmed in a subgroup of Crohn’s disease patients which displayed epigenetic and expression changes in RNF20/40-dependent gene signatures. Our findings reveal that loss of H2B monoubiquitination promotes intestinal inflammation via decreased VDR activity thereby identifying RNF20 and RNF40 as critical regulators of IBD.

## Introduction

Inflammatory bowel disease (IBD), including Crohn’s disease (CD) and ulcerative colitis (UC), affects approximately one individual in 250 Europeans and North Americans [1]. Indeed, rapid increases in the incidence of IBD within populations strongly suggest an environmental component to disease susceptibility. One major link between the exposome and pathophysiology is chromatin modifications stably altering transcriptional programs and cellular function [2]. One such epigenetic modifier, the obligate RNF20/RNF40 heterodimer responsible for H2B monoubiquitination (H2Bub1), has recently been implicated in intestinal inflammation in a context-dependent fashion [3, 4]. A recent CRISPR screen identified RNF20 as a negative modulator of regulatory T cells [5] which play an important role in the pathogenesis of IBD [6, 7]. In addition, the heterozygous global knockout of *Rnf20* in mice resulted in increased infiltration of myeloid-derived suppressor cells [4]. Thus, while the H2B monoubiquitination pathway is clearly essential for immune regulation, little mechanistic detail is known about the cell-intrinsic function of RNF20 and RNF40 in IBD.

Generally, the obligate RNF20/RNF40 heterodimer is recruited by the WW domain-containing adapter protein with coiled-coil (WAC) to the elongating RNA Polymerase II (RNAPII) following phosphorylation of serine 2 within the C-terminal domain of RNAPII [8]. As an E3 ligase complex, the RNF20/40 dimer monoubiquitinates histone H2B at lysine 120, an epigenetic mark associated with increased chromatin accessibility, resulting in eased passage of RNAPII and highly active transcriptional elongation [9]. In addition, as supported by studies in yeast [10, 11] and our own experiments [12], H2Bub1 regulates the trimethylation of histone 3 at lysine 4 (H3K4me3), which is also tightly associated with transcriptional elongation [13]. Epigenetic pathways marked by H3K4me3 occupancy are emerging as relevant therapeutic targets in IBD patients as evidenced by recent studies [14, 15].

Besides its role in transcriptional regulation, H2Bub1 was suggested as a potential tumor-suppressive marker in colorectal cancer (CRC) [16]. Counterintuitively, depletion of RNF20 and RNF40 resulted in reduced tumorigenic potential of CRC cells in vitro, potentially due to enhanced apoptosis rates [3, 17]. In addition, ablation of either, RNF20 or RNF40, modulates Nuclear Factor kappa-light-chain-enhancer of activated B cells (NF-κB) signaling [3, 4, 18]. However, the consequences of *Rnf20* and *Rnf40* loss in the context of intestinal inflammation have not been directly compared in the same experimental settings.

Given the scarce knowledge on the cell-specific functions of RNF20/RNF40, we sought to investigate the importance of the H2B monoubiquitination pathway in IBD through a multidimensional analysis of mouse to human experimental systems. We detected reduced H2Bub1 amounts in 80% of IBD patients and, accordingly, intestinal deletion of *Rnf20* or *Rnf40* resulted in the development of spontaneous colorectal inflammation in mice. Of mechanistic importance, we found *Vitamin D Receptor* (*Vdr)* gene as well as VDR target gene H3K4me3 occupancy and expression to be highly dependent upon RNF20/40. As vitamin D status remains one of the few environmental associations with IBD activity [19], this report provides unique insight into the epigenetic regulation of this key IBD-associated factor.

## Results

### Loss of H2Bub1 is associated with intestinal inflammation in IBD patients

**Fig. 1:**
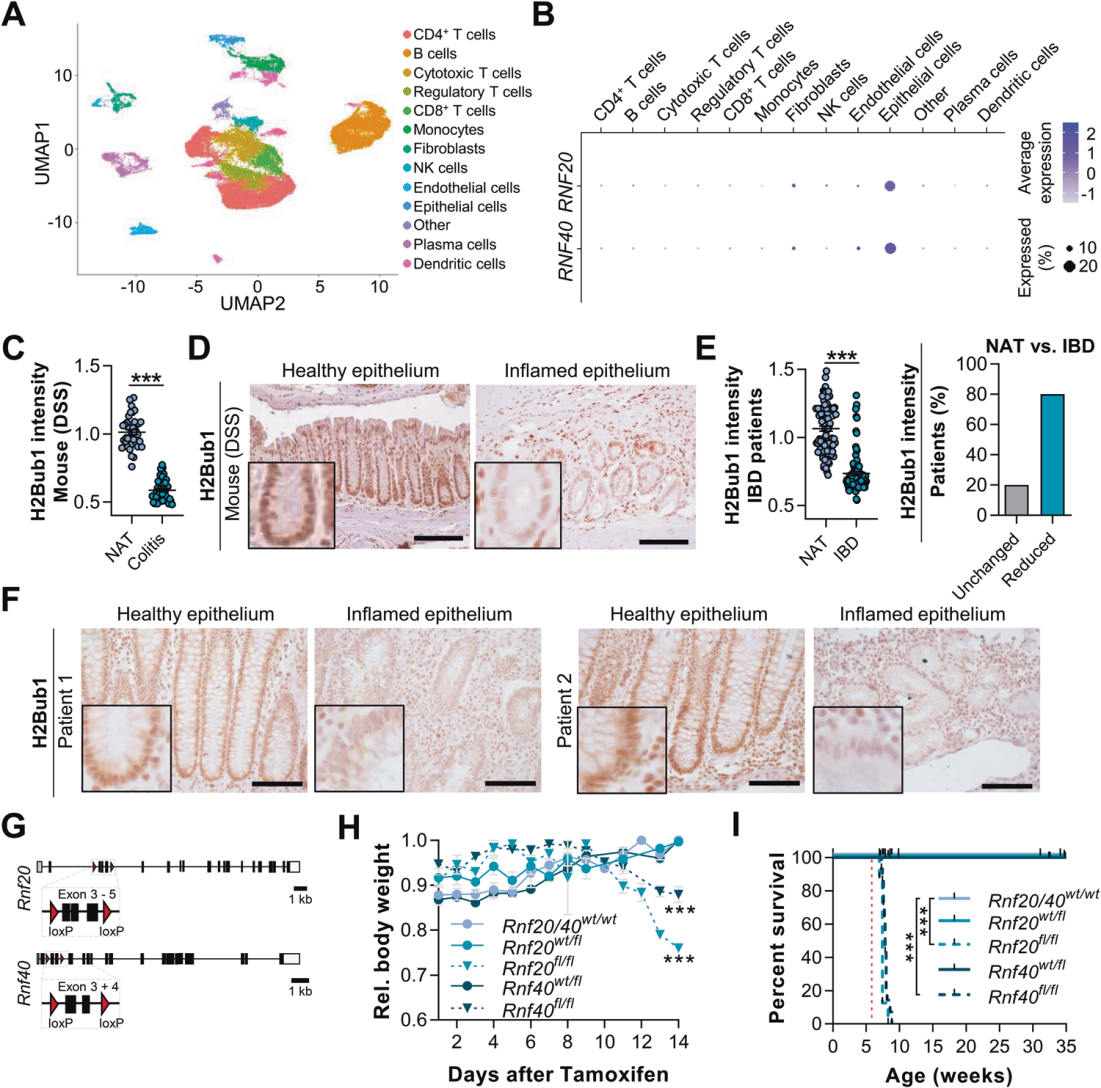
IBD patients display H2Bub1 loss in inflamed intestinal epithelium. **A** Cell type clustering in a uniform manifold approximation and projection (UMAP) based on scRNA-seq data evaluating cell-specific gene expression changes between uninflamed and inflamed resection specimens isolated from Crohn’s disease patients [20]. **B** Dot plot displaying the expression level and the percentage of cells within each cell cluster expressing *RNF20* and *RNF40*. *RNF20* and *RNF40* levels were highest in epithelial cells. **C** Colon sections of C57BL/6N mice treated with 0.75% DSS (*n* = 6) for 14 days were stained for H2Bub1 using IHC. Relative staining intensity was quantified using FIJI in five images per mouse. **D** Representative images of murine colon sections displaying reduced H2Bub1 levels in inflamed epithelium. Scale bar: 100 µm. **E** Intestinal sections of Crohn’s disease patients (*n* = 18) were stained for H2Bub1 using IHC. Relative staining intensity was quantified using FIJI in five images per patient. When comparing healthy resection margins to inflamed tissue, 80% of patients displayed reduced H2Bub1 levels in inflamed regions. **F** Representative images of colon sections of two IBD patients stained for H2Bub1. Scale bar: 100 µm. **G** Mice with loxP-flanked exons 3–5 of *Rnf20* or loxP-flanked exons 3–4 of *Rnf40* were mated with Villin-CreER^T2^ mice to enable a tamoxifen-inducible intestinal knockout of *Rnf20* or *Rnf40*, respectively. **H** Fourteen days after the first tamoxifen injection, *Rnf20*^fl/fl^ and *Rnf40*^fl/fl^ mice displayed severe weight loss and, therefore, (**I**) significantly reduced survival (red dotted line represents start of tamoxifen treatment). Survival data were based on local and institutional guidelines according to which animals needed to be sacrificed after a weight loss of 20%. One-way ANOVA, mean ± SEM.

A previous mouse model of global haploinsufficiency of *Rnf20* suggested that the H2B monoubiquitination pathway may suppress intestinal inflammation [4]. However, there is a lack of knowledge on cell-specific functions of RNF20 and RNF40 in IBD. To examine a potential cell type-specific function of these H2B ubiquitin ligases, we analyzed a recent single cell RNA-seq dataset comparing cell-specific transcriptome-wide effects in Crohn’s disease (CD) patients [20]. Upon clustering of cell types (Fig. 1A), we detected *RNF20* and *RNF40* gene expression primarily within epithelial cells (Fig. 1B). Therefore, to test for a putative function of the RNF20/40 axis in controlling intestinal inflammation, we studied colon sections of dextran sodium sulfate (DSS)-treated mice by performing immunohistochemistry for H2Bub1. Interestingly, H2Bub1 was lost in the majority of inflamed epithelial areas (Fig. 1C, D). To evaluate, whether this finding could be translated to the human condition, we performed immunohistochemical staining of H2Bub1 in a panel of human Crohn’s disease samples with corresponding uninflamed adjacent tissue (NAT) from resection margins as a control. Notably, when quantifying nuclear staining intensity, we observed a significant decrease in H2Bub1 levels in inflamed epithelium (Fig. 1E, F). In fact, when comparing inflamed to healthy tissue within the same individual, we discovered that 80% of IBD patients displayed a strong reduction in H2Bub1-positive inflamed epithelial cells, while only 20% of patients displayed no changes in H2Bub1 levels. The observed association between H2Bub1 deficiency and intestinal inflammation led us to subsequently investigate the in vivo mechanistic role of RNF20/40 in the intestinal epithelium.

### Genetic deletion of *Rnf20* or *Rnf40* promotes spontaneous colorectal inflammation

To evaluate the biological and molecular consequences of H2Bub1 loss in intestinal epithelial cells, we generated Villin-CreER^T2^, *Rnf20*^flox,^ and Villin-CreER^T2^, *Rnf40*^flox^ mice (Fig. 1G). The conditional tamoxifen-inducible deletion of each of the E3 ligases *Rnf20* and *Rnf40* individually provided complementary approaches to induce the loss of H2Bub1 in intestinal epithelial cells. Notably, 14 days after the first tamoxifen injection, *Rnf20*^fl/fl^ and *Rnf40*^fl/fl^ mice already displayed severe weight loss of up to ca. 15% (Fig. 1H, Supplementary Fig. S1A). Accordingly, the Disease Activity Index (DAI) was elevated in these animals (Supplementary Fig. S1B-E). As a consequence of high disease activity, *Rnf20*^fl/fl^ and *Rnf40*^fl/fl^ mice displayed significantly decreased survival compared to wild type and heterozygous litter mates (Fig. 1I).

**Fig. 2:**
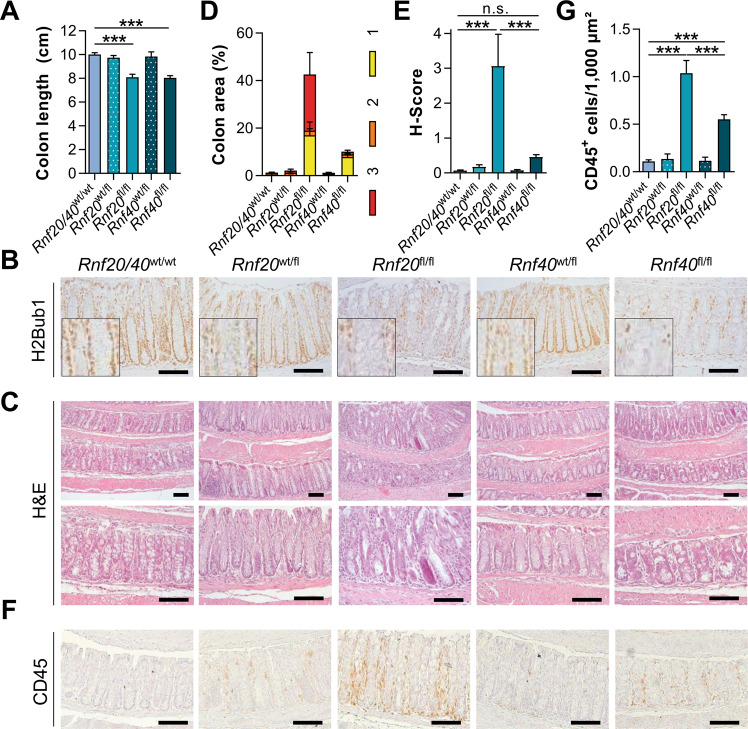
Intestinal *Rnf20* and *Rnf40* deletion results in spontaneous colorectal inflammation in mice. **A** Homozygous *Rnf20* or *Rnf40* deletion resulted in decreased colon length. **B** The knockout efficiency in *Rnf20*^fl/fl^ and *Rnf40*^fl/fl^ mice was demonstrated using IHC for H2Bub1 on colon sections. **C** Colons were H&E-stained and (**D**) the percentage of segments with mild [1], medium [2], or severe [3] epithelial damage was determined. *Rnf20* and *Rnf40* intestine-specific knockout mice displayed more areas with epithelial damage than wildtype or heterozygous animals. **E** Based on the damage, the *H* score was calculated and shown to be elevated upon the loss of *Rnf20* and *Rnf40*. **F** IHC for CD45-positive cells in the colon revealed (**G**) increased infiltration of immunoregulatory cells. Scale bar: 100 µm. One-way ANOVA, mean ± SEM.

Consistent with an increased DAI and reduced survival, mice with an intestinal deletion of *Rnf20* or *Rnf40* displayed significantly decreased colon length (Fig. 2A, Supplementary Fig. S1F, G). After confirming the successful loss of H2Bub1, RNF20, and RNF40 in *Rnf20*^fl/fl^ and *Rnf40*^fl/fl^ animals (Fig. 2B, Supplementary Fig. S1H), H&E staining of the colon revealed severe epithelial damage in *Rnf20-* and *Rnf40-*deficient mice (Fig. 2C). The colorectal epithelial damage was quantified by determination of the H-score, a scoring system based on the intactness of the colonic epithelium and lymphocyte infiltration. Indeed, *Rnf20*^fl/fl^ and *Rnf40*^fl/fl^ mice displayed a high degree of epithelial damage and lymphoid infiltrates, contributing to a significantly increased H-score (Fig. 2D, E; Supplementary Fig. S1I). Consistently, we observed increased infiltration of CD45-positive immune cells (Fig. 2F, G) and increased vascularization (Supplementary Fig. S1J, K) in these animals while only minor effects were observed on serum IL-6 levels (Supplementary Fig. S1L). In summary, the Villin-CreER^T2^-mediated deletion of *Rnf20* or *Rnf40* promotes spontaneous colorectal inflammation in mice. In contrast, mice heterozygous for *Rnf20* or *Rnf40* deletion did not develop spontaneous inflammation (Supplementary Fig. S2) and did not display increased sensitivity to DSS-mediated colitis (Supplementary Fig. S3). These results suggest that only a complete loss of *Rnf20* and *Rnf40*, and therefore H2Bub1, is sufficient to induce spontaneous colorectal inflammation.

### *Rnf20*/*40* deletion promotes IBD-associated gene expression programs

**Fig. 3:**
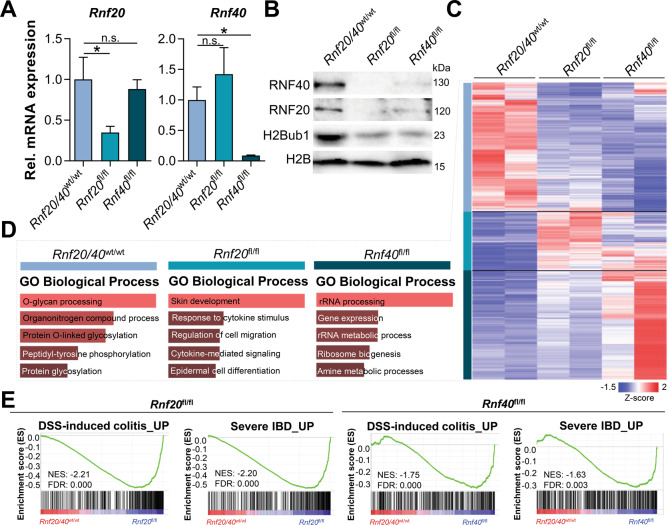
The intestinal loss of either *Rnf20 or Rnf40* drives IBD-associated transcriptome-wide changes. **A** The loss of *Rnf20* or *Rnf40* in IECs was confirmed at the mRNA and (**B**) protein levels using qRT-PCR and western blot, respectively. **C** mRNA-seq was performed using wild type, *Rnf20*^fl/fl^ and *Rnf40*^fl/fl^ IECs (*n* = 2). Heatmap depicting the transcriptome-wide effects of *Rnf20* and *Rnf40* deletion by showing significantly (padj ≤ 0.05) upregulated (log2 FC ≥ 1, red) or downregulated (log2 FC ≤ −1, blue) genes. **D** Based on expression patterns, genes were divided into three clusters which were analyzed using gene ontology (Enrichr) [21, 22]. Genes enriched in wild type controls were associated with O-glycosylation while genes enriched in *Rnf20* and *Rnf40* knockout mice correlated with cytokine-mediated signaling and gene expression processes, respectively. **E** Differentially regulated genes upon *Rnf20* and *Rnf40* knockout in IECs were compared to previously published gene expression signatures [23, 24] using GSEA. Transcriptional changes associated with DSS-induced colitis in mice as well as severe IBD were enriched in *Rnf20*^fl/fl^ and *Rnf40*^fl/fl^ IECs. One-way ANOVA, mean ± SEM.

To gain initial insight into the molecular mechanisms by which perturbation of the H2B monoubiquitination axis leads to intestinal inflammation, we examined transcriptome-wide changes in intestinal epithelial cells (IECs) directly isolated from the colons of Villin-CreER^T2^, *Rnf20*^fl/fl^ and *Rnf40*^fl/fl^ mice and compared these to wild type controls 14 days after tamoxifen injection. After confirming the loss of *Rnf20* and *Rnf40* expression at the mRNA and protein level as well as the loss of H2Bub1 (Fig. 3A, B), we performed mRNA-seq. As expected, *Rnf20* and *Rnf40* deletion resulted in the differential regulation of a broad variety of genes (Fig. 3C). Gene subsets were divided into three clusters (upregulated in wild type, *Rnf20*^fl/fl^ or *Rnf40*^fl/fl^, respectively) and analyzed for pathway enrichment using Enrichr [21, 22]. Interestingly, factors involved in O-glycosylation of mucins and other proteins were commonly downregulated upon loss of *Rnf20* or *Rnf40*. While many processes were commonly enriched following deletion of either H2B ubiquitin ligase, *Rnf20*^fl/fl^ IECs demonstrated a higher enrichment of gene sets related to cytokine signaling and epidermal differentiation while *Rnf40*^fl/fl^ cells showed an enrichment of ribosome biogenesis and gene expression-related processes (Fig. 3D). Finally, we sought to determine whether gene expression patterns altered following *Rnf20* and *Rnf40* deletion in IECs may be relevant for murine models for colitis and human IBD. Indeed, we found that genes related to inflamed tissue in DSS-treated mice [23] as well as in patients with severe IBD [24] were enriched in *Rnf20*^fl/fl^ and *Rnf40*^fl/fl^ IECs (Fig. 3E). Thus, the intestinal deletion of *Rnf20* and *Rnf40* in murine IECs is associated with gene expression profiles induced in murine and human intestinal inflammation.

### RNF20 and RNF40 are required for the expression of IBD susceptibility genes

**Fig. 4:**
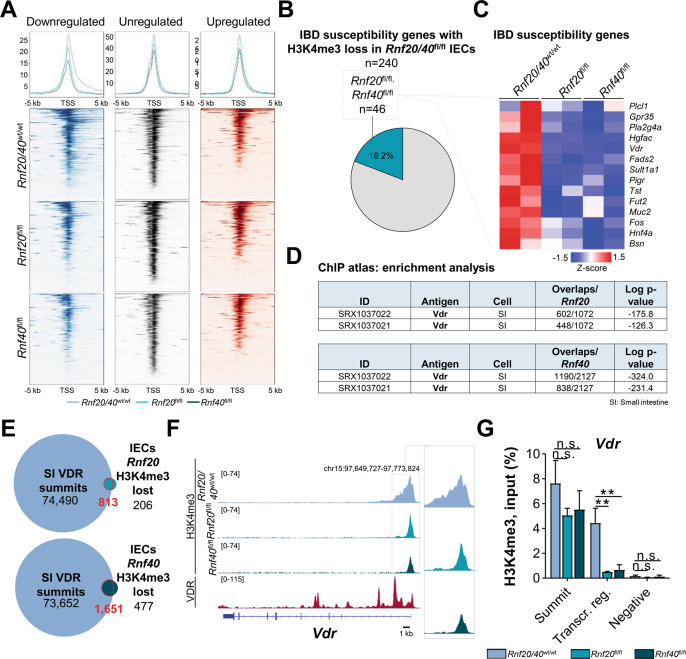
Loss of RNF20 or RNF40 causes reduced H3K4me3 occupancy on IBD risk genes including *Vdr*. **A** Average binding profiles and heatmaps depicting occupancy (RPKM values) of H3K4me3 around the TSS ( ± 5 kb) of genes upregulated, unregulated and downregulated in wild type, *Rnf20*^fl/fl^ and *Rnf40*^fl/fl^ IECs. Genes were sorted high to low based on H3K4me3 occupancy under wild type conditions. Binding profiles revealed decreased H3K4me3 occupancy specifically in the gene body after *Rnf20* or *Rnf40* loss leading to peak narrowing. **B** Genes which lost H3K4me3 occupancy in *Rnf20*^fl/fl^ and *Rnf40*^fl/fl^ IECs were compared for overlap with IBD susceptibility genes [27, 28] expressed in IECs. H3K4me3 occupancy of 19% of IBD risk genes is dependent on RNF20 and/or RNF40. **C** Heatmap depicting gene expression patterns of a subset of IBD susceptibility genes in wild type, *Rnf20*^fl/fl^ and *Rnf40*^fl/fl^ IECs (padj ≤ 0.05, red: upregulated (log2 FC ≥ 1), blue: downregulated (log2 FC ≤ −1)). **D** Regions that lost H3K4me3 occupancy in *Rnf20* and *Rnf40* null IECs were compared to previously published ChIP-seq experiments using ChIP Atlas (http://chip-atlas.org) displaying a high overlap between our datasets and VDR-bound regions in the murine small intestine (SI) [29]. **E** By analyzing publicly available VDR ChIP-seq data, VDR-bound regions in murine SI were identified (75,303 regions) [29] and compared to regions displaying decreased H3K4me3 occupancy in *Rnf20* (1,019 regions) and *Rnf40* (2,128 regions) knockout IECs. 79.8% (813/1,019 regions) of *Rnf20*- and 77.6% (1,651/2,128 regions) of *Rnf40*-dependent H3K4me3 regions were also occupied by VDR. **F** Binding profiles of VDR in SI [29] and H3K4me3 in IECs revealed H3K4me3 peak narrowing under *Rnf20* or *Rnf40* knockout conditions. **G** Reduced H3K4me3 occupancy in the *Vdr* gene body in *Rnf20*^fl/fl^ and *Rnf40*^fl/fl^ IECs was confirmed by ChIP-qPCR (*n* = 4). One-way ANOVA, mean ± SEM.

Early studies exploring the molecular function of H2Bub1 revealed that this epigenetic mark is highly enriched across the transcribed region of active genes [25]. Mechanistically, H2Bub1 enables RNAPII passage through chromatin [26]. H2Bub1 also facilitates deposition of H3K4me3 in yeast [10, 11]. The histone modification H3K4me3 is an established mark of actively transcribed transcription start sites (TSS) and gene bodies. Indeed, our prior work substantiated a role for H2Bub1 in elongation-associated spreading of H3K4me3 into the gene body of RNF40-dependent genes [12]. Thus, we performed ChIP-seq in IECs to analyze H3K4me3 occupancy upon the deletion of *Rnf20* and *Rnf40*. Consistent with our prior work, loss of *Rnf20* or *Rnf40* resulted in a significant reduction of H3K4me3 occupancy in the gene body with only marginal effects closer to the TSS (Fig. 4A). Focusing on this discovery, we closely examined the genes displaying RNF20/40-dependent changes in H3K4me3 occupancy and decreased expression. Given the inflammatory consequences of an intestine-specific *Rnf20/40* deletion in mice, we performed an enrichment analysis of the RNF20/40-dependent genes with published IBD susceptibility gene lists [27, 28]. Remarkably, 19% of published IBD susceptibility genes appeared to be regulated by this RNF20/RNF40/H3K4me3-dependent mechanism (Fig. 4B). Consistently, a downregulation of those IBD susceptibility genes at the mRNA level could be confirmed in our mRNA-seq data (Fig. 4C). In summary, these findings reveal a previously unknown function of RNF20/40 in regulating H3K4me3 occupancy and gene expression of a high proportion of IBD susceptibility genes.

### H3K4me3 occupancy on *Vdr* and VDR target genes is RNF20- and RNF40-dependent

**Fig. 5:**
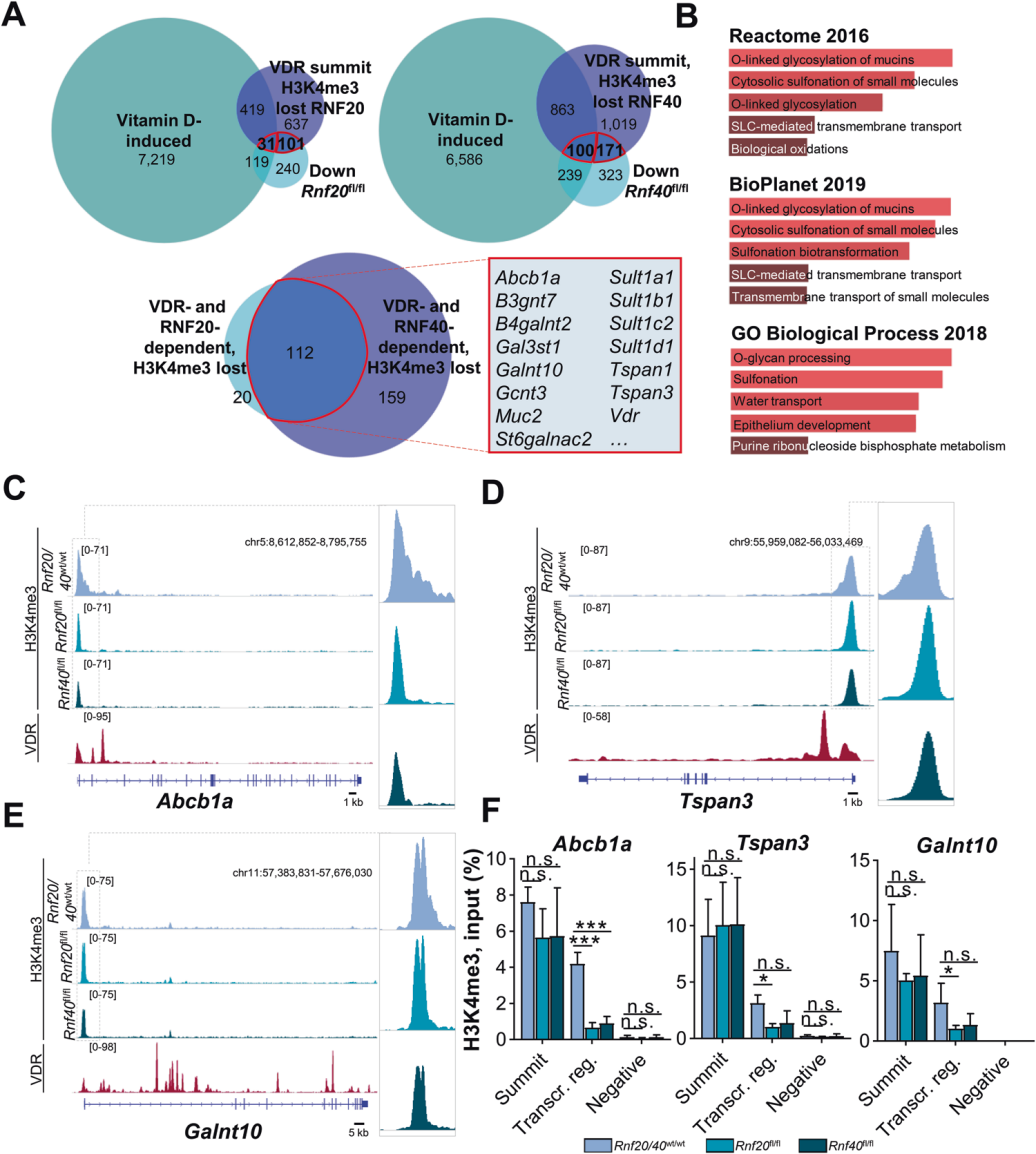
RNF20 and RNF40 are required for transcriptional elongation-associated H3K4me3 spreading on VDR target genes. **A** Venn diagram displaying the overlap between vitamin D-induced genes [29], genes downregulated following *Rnf20* or *Rnf40* knockout in IECs identified by mRNA-seq and VDR-dependent genes displaying reduced H3K4me3 occupancy upon *Rnf20* or *Rnf40* deletion in IECs (upper panel). Numbers marked in red were overlapped to identify VDR- and RNF20/40-dependent genes with reduced H3K4me3 occupancy (lower panel). **B** Enrichr gene ontology analysis of these 112 genes revealed their involvement in O-linked glycosylation and sulfonation of proteins. **C**–**E** ChIP-seq tracks of three exemplary genes (*Abcb1a*, *Tspan3*, *Galnt10*) illustrated H3K4me3 peak narrowing in *Rnf20* and *Rnf40* knockout IECs and VDR occupancy in murine small intestine [29]. **F** ChIP-qPCR for *Abcb1a*, *Tspan3* and *Galnt10* was performed using primers complementary to the peak summit, in the transcribed region and a negative control site. Upon *Rnf20* and *Rnf40* deletion, occupancy in the transcribed region was significantly decreased (*n* = 4). One-way ANOVA, mean ± SEM.

In order to uncover further upstream regulatory mechanisms controlling the expression of RNF20/40-dependent genes in IEC, we examined whether specific transcription factors displayed a particular enrichment on these genes using occupancy data from publically available ChIP-seq datasets using ChIP Atlas (http://chip-atlas.org). Intriguingly, we found Vitamin D Receptor (VDR)-bound regions [29] (small intestine, mouse, Fig. 4D, E) to be significantly enriched within our dataset. Validating our bioinformatic methodology, the *Vdr* gene itself was bound by VDR and displayed profound H3K4me3 peak narrowing following the loss of *Rnf20* or *Rnf40* (Fig. 4F). This finding was further confirmed using ChIP-qPCR, where a significant reduction of H3K4me3 occupancy was observed in the transcribed region of the *Vdr* gene (Fig. 4G). To obtain further insight into the relationship between RNF20/40- and VDR-dependent gene regulation, genes induced in the small intestine of vitamin D-treated mice [29] were overlapped with genes downregulated upon *Rnf20* or *Rnf40* loss (mRNA-seq) and genes which displayed decreased H3K4me3 occupancy in *Rnf20*^fl/fl^ and *Rnf40*^fl/fl^ IECs (ChIP-seq). Genes commonly regulated by vitamin D, RNF20, and RNF40 included several IBD susceptibility genes as well as mucin-modifying factors (Fig. 5A, B). Indeed, ChIP-seq tracks (Fig. 5C-E) and ChIP-qPCR (Fig. 5F) confirmed that these genes displayed reduced H3K4me3 occupancy specifically in their transcribed regions. Together, using corroborating methodology, these results indicate that RNF20 and RNF40 loss reduces H3K4me3 occupancy on the IBD susceptibility gene *Vdr* as well as VDR target genes.

### The IBD risk gene *Vdr* is downregulated upon *Rnf20* or *Rnf40* deletion

**Fig. 6:**
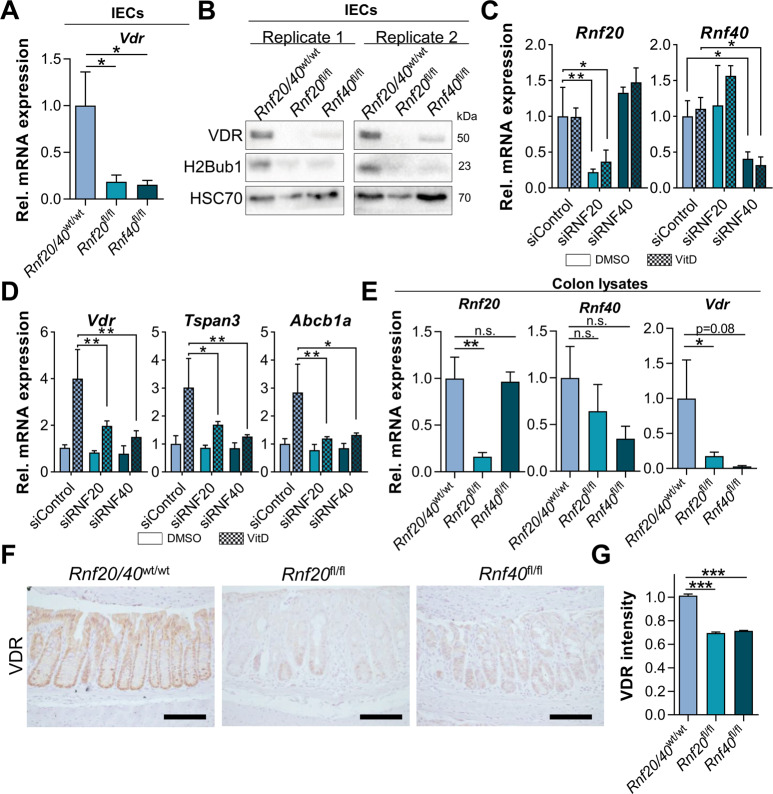
RNF20 and RNF40 deficiency results in reduced VDR levels. **A** *Rnf20* and *Rnf40* deletion reduced *Vdr* mRNA and (**B**) protein levels in IECs as demonstrated by qRT-PCR and Western blot. **C** IECs were isolated from wild type mice and cultured under vitamin D-deficient conditions. RNF20 and RNF40 were transiently depleted using siRNA and 24 h after knockdown cells were treated with calcitriol (1 µM) for 48 h (*n* = 3). **D** Calcitriol treatment induced the mRNA expression of VDR target genes in control cells but not in RNF20- and RNF40-depleted IECs as detected using qRT-PCR. **E** The reduction of *Vdr* following *Rnf20* and *Rnf40* genetic deletion was confirmed in whole colon lysates using qRT-PCR (*n* = 6). **F** IHC for VDR and subsequent (**G**) determination of staining intensity using FIJI verified reduced VDR protein amounts in *Rnf20*^fl/fl^ and *Rnf40*^fl/fl^ colons. Scale bar: 100 µm. One-way ANOVA, mean ± SEM.

We observed that the IBD susceptibility gene *Vdr* encoding the vitamin D receptor was downregulated in our mRNA-seq data and, indeed, we confirmed this finding in IECs at the mRNA and protein levels (Fig. 6A, B). Accordingly, the expression of VDR target genes was also significantly reduced in *Rnf20*^fl/fl^ and *Rnf40*^fl/fl^ IECs (Supplementary Fig. S4A). Notably, the overexpression of human *VDR* in IECs rescued the downregulation of *Vdr* as well as VDR-dependent genes upon the depletion of RNF20 and RNF40 (Supplementary Fig. S4B-C). Thus, multiple independent methodologies support that RNF20/40 regulates *Vdr* and VDR gene targets by modulating H3K4 trimethylation and target gene transcription.

Next, we directly tested this mechanism through a reductionist in vitro approach. As our results were based on a vitamin D-proficient mouse model, we aimed to verify our hypothesis that RNF20 and RNF40 are required to promote expression of *Vdr* and VDR target genes in another system. Thus, we isolated IECs from wild type animals and grew them in 2D cell culture under vitamin D-deficient conditions. Cells were transfected with siRNAs to transiently deplete RNF20 and RNF40 and were treated with calcitriol, the active form of vitamin D. Notably, the induction of *Vdr* and VDR target gene expression upon vitamin D treatment was significantly impaired following RNF20 or RNF40 depletion (Fig. 6C, D). We further confirmed the reduction of *Vdr* and VDR target genes in whole colon lysates isolated from *Rnf20*^fl/fl^ and *Rnf40*^fl/fl^ mice at the mRNA level (Fig. 6E, Supplementary Fig. S5A). Finally, we were able to verify that VDR protein levels and those of one of its targets were decreased upon *Rnf20* and *Rnf40* knockout via IHC on colon sections (Fig. 6F, G, Supplementary Fig. S5B, C).

### A subgroup of CD patients displays deregulation of RNF20/40-dependent gene signatures

**Fig. 7:**
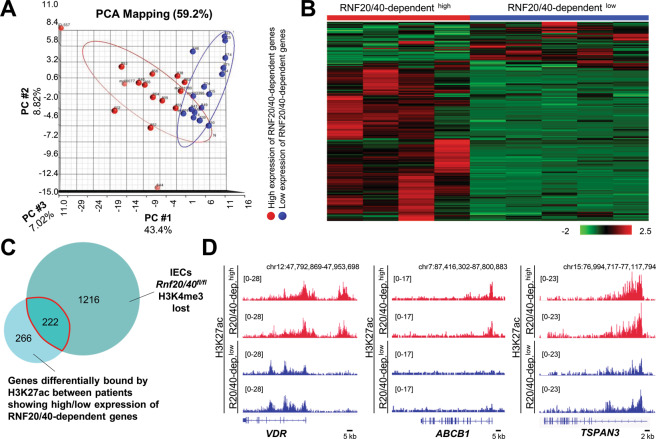
RNF20/40-dependent genes are deregulated in a subset of IBD patients. **A** Principal component analysis demonstrating the separation of IBD patients (*n* = 30) displaying low (in blue) or high (in red) expression of RNF20/40-regulated genes detected by mRNA-seq in murine IECs. **B** Heatmap displaying the expression of genes differentially marked with H3K27ac between a subset of IBD patients with markedly low (*n* = 5) or high (*n* = 4) expression of RNF20/40-dependent genes. **C** Venn diagram showing the overlap between genes differentially bound by H3K27ac in IBD patients showing low or high expression of RNF20/40-regulated genes and genes which have lost H3K4me3 occupancy in *Rnf20*/*40*^fl/fl^ IECs. **D** Example occupancy profiles of H3K27ac on *VDR*, *ABCB1,* and *TSPAN3* revealed decreased H3K27ac occupancy in enhancer and promoter regions in IBD patients with low (blue) compared to high (red) expression of RNF20/40-dependent genes.

We have demonstrated that intestinal *Rnf20* and *Rnf40* deletion results in spontaneous colorectal inflammation in mice as well as reduced levels of *Vdr* and its target genes. Given the established association of Vitamin D biology as a risk factor to Crohn’s disease activity, a cohort of CD patients was analyzed for gene expression and H3K27ac occupancy. We utilized an emerging methodology of RNA/chromatin extraction from FFPE tissue from terminal ileal resection specimens isolated from a well characterized cohort of CD patients. Confirming the importance of this pathway, a subgroup of individuals displayed consistently low expression for the majority of genes downregulated in *Rnf20*^fl/fl^ and *Rnf40*^fl/fl^ IECs (Fig. 7A, B). Next, genes differentially marked with H3K27ac between patients with low and high expression of RNF20/40-dependent genes were overlapped with genes which lost H3K4me3 occupancy in *Rnf20*^fl/fl^ and *Rnf40*^fl/fl^ IECs. Intriguingly, 45% of genes differentially marked by H3K27ac in IBD patients also displayed decreased H3K4me3 in *Rnf20/40*-deficient IECs (Fig. 7C). Indeed, patients with low expression of RNF20/40-dependent genes displayed reduced H3K27ac occupancy on the IBD risk gene *VDR* as well as VDR targets such as *ABCB1* and *TSPAN3* (Fig. 7D). Notably, these patient subsets did not display differences in serum vitamin D levels, similar to the situation observed in our mouse model (Supplementary Fig. S5D, E). Together, our findings demonstrate that RNF20 and RNF40 not only regulate the IBD susceptibility gene *VDR* but also a high proportion of VDR target genes in mice. Importantly, we identified a subset of IBD patients that displays deregulated expression and epigenetic marking of RNF20/40-dependent VDR targets.

## Discussion

Our findings uncover a novel role for RNF20 and RNF40 in maintaining *Vdr* and VDR target gene expression and suppression of intestinal inflammation. While decreased H2Bub1 levels have been linked to tumor progression and poor survival in several cancer entities, including CRC [16], we report for the first time that H2B monoubiquitination is reduced in inflamed areas in 80% of Crohn’s disease patients. In support of this finding, we observed that complete intestine-specific ablation of *Rnf20* or *Rnf40*, the genes encoding the E3 ligases mediating H2B monoubiquitination, promotes spontaneous intestinal inflammation in mice. Molecularly, our findings demonstrate that RNF20/40 loss leads to a global narrowing of H3K4me3 peaks, especially on VDR-dependent genes.

Vitamin D is thought to be an important factor in the development and perpetuation of IBD, particularly CD [30]. For example, vitamin D deficiency in IBD patients has been described in several studies and was associated with increased disease activity [19]. These findings were confirmed in an *Il10* knockout-based experimental model for murine colitis and, indeed, vitamin D supplementation ameliorated disease activity and reduced mortality [31]. The biological activity of vitamin D is mediated by the binding of its active metabolite 1,25-dihydroxyvitamin D3 (1,25(OH)2D3; calcitriol) to the vitamin D receptor [32]. Intriguingly, *VDR* gene polymorphisms have also been described to increase IBD susceptibility [33, 34] and VDR levels were found to be profoundly reduced in inflamed colonic biopsies isolated from IBD patients compared to healthy controls. Accordingly, the deletion of *Vdr* in gut epithelial cells resulted in severe intestinal inflammation in chemically induced colitis in mice while elevated *Vdr* expression protected animals from symptoms [35]. Despite these findings supporting an important role of vitamin D receptor activity in IBD, environmental or epigenetic modifiers regulating this pathway remained to be elucidated.

As originally shown in yeast [10, 11] and supported by our previous studies [12], H2B monoubiquitination regulates H3K4me3, an epigenetic mark associated with increased transcription elongation rates [13]. Consistently, the loss of H2Bub1 following intestinal *Rnf20* or *Rnf40* deletion resulted in decreased H3K4me3 occupancy in the transcribed region of various IBD-associated genes. Notably, upon H2Bub1 loss, we observed H3K4me3 narrowing on the *Vdr* gene as well as decreased *Vdr* mRNA and protein amounts. Consequently, the induction of VDR-dependent gene expression was also impaired. Several of these common RNF20/RNF40/VDR targets were shown to be downregulated in experimental colitis, such as *Muc2* [36]. This finding is in agreement with our recent publication describing that H2Bub1 controls early stages of osteoblast differentiation and bone cell crosstalk by regulating the expression of the VDR target gene *Tnfsf11* [37]. In general, the finding that VDR-dependent genes are frequently downregulated in IBD supports vitamin D supplementation in clinical practice as well as experimental trials. Whether the presence of H2Bub1 may potentially serve as a predictive biomarker for vitamin D responsiveness in IBD patients remains to be elucidated. However, the observation that neither *Rnf20/40*-deficient mice, nor patients with low expression of RNF20/40-dependent genes displayed vitamin D deficiency suggests that vitamin D supplementation alone would likely not be sufficient to compensate for decreased vitamin D receptor levels.

Despite the common deregulation of *Vdr* and VDR target genes, our mRNA-seq data demonstrate that some genes are differentially affected by the deletion of *Rnf20* and *Rnf40* in IECs. This finding suggests that RNF20 and RNF40 may have additional, unique ubiquitination targets in addition to H2B involved in the regulation of gene expression. Future mass spectrometry-based identification of ubiquitinated proteins in the presence or absence of RNF20/40 is needed to clarify the distinct functions of RNF20 and RNF40.

Interestingly, the role of H2Bub1 in controlling intestinal inflammation appears to be dramatically different depending upon the degree to which it is lost. In a previous study, we employed a different conditional knockout model for the distal intestinal epithelium (CAC-Cre) which results in a  low knockout efficiency [38]. Using this model to delete *Rnf40* resulted in a sporadic loss of RNF40 and H2Bub1 staining in only 15% of colorectal epithelial cells and paradoxically elicited a protective effect during DSS-induced colitis. This finding is in agreement with the current study in which the inefficient reduction of *Rnf20* or *Rnf40* in heterozygous animals did not promote colorectal inflammation. Notably, when evaluating why heterozygous mice only displayed minor differences when compared to wild-type controls, we detected that H2Bub1 staining intensity was similar across wild-type and heterozygous genotypes. Thus, we hypothesize that only a complete loss of H2Bub1 in the intestinal epithelium is sufficient to elicit spontaneous colitis.

The H2B monoubiquitination pathway likely plays multiple roles in controlling IBD in different cell types. Interestingly, a recent CRISPR screen identified RNF20 as a negative regulator of the master regulatory T cell (Treg) transcription factor Forkhead box P3 (FOXP3) [5]. In fact, FOXP3-positive Tregs play an important role in IBD [6, 7]. Similar divergent cell type-dependent differential functions of an epigenetic regulator in IBD have been demonstrated for the Polycomb Repressive Complex-2 methyltransferase component EZH2. In this case, while global inhibition of EZH2 activity or myeloid-specific *Ezh2* deletion ameliorates experimental intestinal inflammation [39] the loss of *Ezh2* in either the intestinal epithelium [15] or regulatory T cells [7] results in a spontaneous colitis phenotype. Thus, when considering the H2B monoubiquitination pathway as a potential therapeutic target in IBD, it will be essential to determine and consider the differential roles it plays in diverse cell types important for IBD biology.

In conclusion, this work describes a previously unknown role of RNF20/40-mediated H2B monoubiquitination in the establishment of broad H3K4me3 domains, which appear to promote the transcriptional elongation of VDR-dependent genes associated with intestinal inflammation. In vivo experiments revealed that H2Bub1-deficient mice developed spontaneous colorectal inflammation and, in support of these findings, we detected that 80% of IBD patients displayed a loss of H2Bub1 in inflamed areas. Notably, we detected a subgroup of IBD patients who display deregulated expression and epigenetic control of RNF20/40-dependent gene signatures affecting *VDR* and VDR target genes. Based on this, additional studies will be essential to uncover the interdependence of VDR activity and H2B monoubiquitination and determine the potential therapeutic utility of targeting these for the benefit of patients with IBD.

## Methods

### Patient work

To evaluate H2Bub1 levels in IBD sections, resection specimens of 18 Crohn’s disease patients (Supplementary Table S1) were analyzed with approval of the local ethics committee of the University Medical Center Goettingen, Germany (registration number: 1/10/19). For each patient, inflamed epithelium, as well as healthy resection margins, were assessed. Moreover, H3K27ac occupancy was analyzed in resection specimens of 30 Crohn’s disease patients using chromatin immunoprecipitation (ChIP) as approved by the local ethics committee of the Mayo Clinic, Rochester, MN, USA (Supplementary Table S2; registration number: IRB #18-002942). All research was performed in accordance with the principles expressed in the Declaration of Helsinki, all study participants voluntarily participated in the study and gave informed consent.

### Animal work

The *Rnf20*^tm1c(EUCOMM)Wtsi^ mouse line, referred to here as *Rnf20*^flox^, containing loxP sites flanking exons 3–5 was generated from the mouse embryonic stem cell line G11 (EPD0701_3_G11) purchased from the European Conditional Mouse Mutagenesis Program (EUCOMM) by crossing “knockout first” allele (*Rnf20*^tm1a(EUCOMM)Wtsi^) mice with a mouse line ubiquitously expressing the Flp recombinase (C57BL/6 N(Cag-FlpO)1Afst/Mmcd) [40]. The *Rnf40*^flox^ mouse line with loxP sites flanking exons 3–4 was described previously[12]. Both *Rnf20*^flox^ and *Rnf40*^flox^ lines were crossed with Villin-CreER^T2^ [41] in a C57BL/6 N background to achieve tamoxifen-inducible intestinal deletion of floxed *Rnf20* or *Rnf40* alleles. For knockout induction, mice were intraperitoneally injected with a dose of 1 mg tamoxifen (Sigma-Aldrich Co., St. Louis, USA) per day for 5 consecutive days at the age of 6 weeks. Numbers of mice sacrificed 14 days after the first tamoxifen injection were *n* = 10 (*Rnf20/40*^wt/wt^), *n* = 7 (*Rnf20*^wt/fl^), *n* = 8 (*Rnf20*^fl/fl^), *n* = 7 (*Rnf40*^wt/fl^), *n* = 17 (*Rnf40*^fl/fl^) and mice observed over a period of 6 months *n* = 8 (*Rnf20/40*^wt/wt^), *n* = 10 (*Rnf20*^wt/fl^), *n* = 7 (*Rnf40*^wt/fl^). To induce acute colitis, animals were treated with 0.75% dextran sodium sulfate (DSS; MO Biomedicals, LLC, Illkirch, France) for 14 consecutive days as previously described [3] (*n* = 9 (*Rnf20/40*^wt/wt^), *n* = 10 (*Rnf20*^wt/fl^), *n* = 7 (*Rnf40*^wt/fl^)).

### Mouse genotyping

DNA was isolated using a Mouse Direct PCR Kit (Stratech Scientific, Ely, UK). Upon an initial denaturation step at 94 °C for 2 min, DNA fragments were amplified as follows: 34 cycles of 94 °C for 20 s, 58 °C for 30 s and 72 °C for 1 min (*Rnf20*); 34 cycles of 94 °C for 20 s, 62 °C for 30 s and 72 °C for 1 min (*Rnf40*); 34 cycles of 94 °C for 20 s, 56 °C for 30 s and 72 °C for 1 min (Cre-recombinase) with a final elongation at 72 °C for 5 min. Primer sequences are listed in Supplementary Table S3. Fragments were subsequently analyzed by agarose gel electrophoresis.

### Determination of the Disease Activity Index (DAI)

To monitor health condition of mice, the DAI was calculated as previously described [3]. Briefly, the DAI includes scores for weight loss (0: 0–1%; 1: 1–5%; 2: 5–10%; 3: 10–15%; 4: >15%), stool consistency (0: normal; 1: soft; 2: very soft; 3: diarrhea) and the presence of occult blood as determined using the stool guaiac test (0: no blue staining; 1: weak/sporadic staining; 2: medium staining; 3: strong blue staining; 4: bloody anus). Investigators were blinded during mouse handling including the determination of the disease activity index.

### Isolation of intestinal epithelial cells (IECs)

Upon sacrificing Villin-CreER^T2^, *Rnf20/40*^wt/wt^, *Rnf20*^fl/fl^ and *Rnf40*^fl/fl^ mice fourteen days upon the first tamoxifen injection, colons were isolated (*n* = 3 pooled/genotype) and flushed with ice-cold PBS. Colons were opened longitudinally and washed in ice-cold PBS. A total of <1 mm² fragments were incubated with collagenase type I (GIBCO^®^, Invitrogen GmbH, Darmstadt, Germany) solution 2 mg/ml collagenase in washing medium (DMEM/F12 + HEPES supplemented with 10% fetal bovine serum (FBS), 100 units/ml penicillin, and 100 μg/ml streptomycin) at 37 °C for approximately 30–40 min. Suspensions were pipetted vigorously up and down until 50–80% of the crypts were separated from the muscle layer. Next, suspensions were transferred to tubes containing 10 ml washing medium. Upon centrifugation at 250 x g for 5 min, supernatants containing muscle fragments were aspirated and the cell pellets were re-suspended in 10 ml washing medium. For subsequent RNA, ChIP and protein analyses, IEC pellets were snap-frozen in liquid nitrogen and stored at −150 °C. For cell culture experiments, wild type IECs were resuspended in growth medium.

### IEC cell culture and transfections

IECs were grown in Dulbecco’s Modified Eagle Medium (DMEM) supplemented with 20% FBS, 100 units/ml penicillin and 100 μg/ml streptomycin at 37 °C and 5% CO_2_. To transiently deplete RNF20 and RNF40, siRNA (GE Dharmacon siGENOME; non-targeting siRNA 5 [D-001210-05-20], RNF20 siRNAs (D-041733-01, −02, −03, −04), RNF40 siRNAs (D-059014-01, −02, −03, −04)) transfections were performed using Lipofectamine^®^ RNAiMAX (Invitrogen GmbH, Karlsruhe, Germany) according to the manufacturer’s instructions. After 24 h, cells were treated with 1 μM calcitriol (Biomol, Hamburg, Germany), the active form of vitamin D, dissolved in DMSO or DMSO alone for 48 h. For *VDR* overexpression, IECs were transfected with a VDR-eGFP expression plasmid [42] or a control plasmid using Lipofectamine^®^ 2000 (Invitrogen GmbH, Karlsruhe, Germany) according to the manufacturer’s instructions. Plasmids were a kind gift from Rajiv Kumar and Theodore A. Craig (Department of Internal Medicine, Division of Nephrology and Hypertension, Mayo Clinic, Rochester, MN). Forty-eight hours after transfection, siRNAs were added and 72 hours after knockdown, RNA was isolated. Overexpression of *VDR* was verified by qRT-PCR and subsequent agarose gel electrophoresis.

### Histology and determination of the Histo-score (H-score)

Hematoxylin and Eosin (H&E) staining and immunohistochemical (IHC) staining of paraffin sections were performed as previously described [3, 43]. Antibodies utilized for IHC are listed in Supplementary Table S4. To determine inflammation intensity, the H-score was determined on H&E-stained colon sections as previously described [3]. It ranges from 0 to 3, considering normal epithelium (0), as well as intestinal areas displaying mild [1], medium [2], or severe [3] epithelial damage. Investigators were blinded during the analysis of the H-score and IHC stainings.

### Western blotting and quantitative real-time PCR (qRT-PCR)

Protein isolation, western blot analysis, RNA extraction, reverse transcription, and qRT-PCR were performed as previously described [17]. Gene expression levels were assessed and normalized to the housekeeping gene *Rplp0*. Primary antibodies for western blot and primers for qRT-PCR are listed in Supplementary Tables S4 and S5, respectively.

### mRNA-seq and analysis

mRNA sequencing libraries were prepared using the TruSeq RNA Library Prep V2 kit (Illumina^®^) and sequencing was performed on a HiSeq4000 (Illumina^®^) at the Genome Analysis Core, Mayo Clinic as reported earlier [43]. Fastq files were mapped to the mouse reference genome mm9 and analysis was performed as previously described [43]. All data have been deposited at ArrayExpress (http://www.ebi.ac.uk/arrayexpress, accession number: E-MTAB-9405). Gene set enrichment analysis (GSEA) [44, 45] was performed using custom gene sets containing genes significantly regulated in mice undergoing acute DSS-induced colitis [23] and in inflamed intestinal biopsies isolated from patients displaying severe IBD [24]. In addition, genes differentially regulated in small intestines (SI) of vitamin D-treated mice [29] were compared to our own data.

### Chromatin immunoprecipitation (ChIP) and ChIP-seq

Snap-frozen IECs were thawed on ice, resuspended in 8 ml of 1% formaldehyde in PBS and incubated at room temperature on a rolling device for 20 min. ChIP was performed as previously described [17].

Chromatin preparation from formalin-fixed paraffin-embedded (FFPE) resection specimens from 30 Crohn’s disease patients was performed following the published ChromEX-PE technology [46] with slight modifications. Briefly, two 20 µm sections were deparaffinized and rehydrated. Tissues were incubated in 500 µl of chromatin stabilization buffer (10 mM Tris-HCl, pH 7.5, 10 mM NaCl, 10 mM EDTA, 0.5% Triton X-100, 0.1% sodium deoxycholate, 20% EtOH) at 65 °C overnight and centrifuged at 21,130 x g at 4 °C for 5 min. Tissue pellets were resuspended in 250 µl RIPA buffer (10 mM Tris-HCl, pH 8.0, 140 mM NaCl, 1 mM EDTA, 1% Triton X-100, 0.1% SDS) and lysates were sonicated (20 cycles, 30 sec on/off) using a Bioruptor Pico sonicator (Diagenode, Inc., Denville, NJ). Supernatants (chromatin inputs) were collected after centrifugation (21,130x*g* at 4 °C for 5 min) and were incubated with 0.2 µg anti-H3K27ac antibody overnight. After adding 15 µl of protein G-magnetic beads, the reactions were further incubated for 3 hours. The beads were extensively washed with ChIP buffer, high salt buffer, LiCl_2_ buffer, and TE buffer. Bound chromatin was eluted and reverse-crosslinked at 65 °C overnight. DNA was purified using the MinElute PCR purification kit (Qiagen, Valencia, CA) after treatment with RNase A and proteinase K. Antibody information is listed in Supplementary Table S4 and ChIP-qPCR primers in Supplementary Table S6.

IEC ChIP libraries were prepared using the KAPA Hyper Prep Kit (KAPABiosystems, Cape Town, South Africa) and H3K27ac libraries (patient samples) using the ThruPLEX^®^ DNA-seq Kit V2 (Rubicon Genomics, Ann Arbor, MI, USA). Library quality was estimated using an Agilent Bioanalyzer 2100 and samples were sequenced (single-end 50 bp) on a HiSeq4000 (Illumina^®^, Genome Analysis Core, Mayo Clinic). Reads were mapped to the murine reference genome mm9 and analysis was performed as previously described [17]. Occupancy profiles were viewed using Integrative Genomics Viewer (IGV 2.5.0) [29]. In addition, to evaluate VDR occupancy, previously published ChIP-seq data [28] have been compared to our own sequencing results. H3K27ac libraries were prepared using the ThruPLEX^®^ DNA-seq Kit V2 (Rubicon Genomics, Ann Arbor, MI, USA) and sequenced to 51 base pairs from both ends on a HiSeq4000 (Illumina^®^) in the Mayo Clinic Center for Individualized Medicine Medical Genomics Facility. The enrichment was analyzed by targeted real-time PCR in positive and negative genomic loci. H3K27ac ChIP-seq data were processed using the internal HiChIP pipeline to generate library-size normalized signal tracks for visualization and a list of peaks [30]. Briefly, paired-end reads were mapped to the human reference genome (hg38) by Burrows-Wheeler Alignment [31] with default settings and only pairs with at least one of the ends being uniquely mapped were retained for further analysis. Alignments were position sorted and duplicates were removed using Picard tools (https://broadinstitute.github.io/picard/). Peaks were called using the MACS2 algorithm at FDR ≤ 1%. Differential binding peaks for patients with RNF20/40-associated gene signatures were called using the DiffBind R package [32]. Peaks with *p* value <1e-5 and absolute log2 fold change >1 were retained as significant. ChIP-seq data have been deposited at ArrayExpress (http://www.ebi.ac.uk/arrayexpress, accession E-MTAB-9409).

### Single cell RNA-seq (scRNA-seq)

Publically available single cell RNA-seq data based on uninflamed and inflamed intestinal resection specimens isolated from Crohn’s disease patients (*n* = 11) were analyzed as previously described [20]. Briefly, sequencing data were aligned to the human reference genome Grch38, and data with at least 500 unique molecular identifiers (UMIs), log10 genes per UMI >0.8, >250 genes per cell and a mitochondrial ratio of less than 0.2% were extracted, normalized and integrated using the seurat package v3.0 in R 4.0.2.

### Statistics

Graphs were generated with GraphPad Prism version 8.0.1 (GraphPad Software, Inc.), R version 3.6.2 or 4.0.2 and Partek^®^ Genomics Suite^®^ software version 7.0 (Copyright^©^ 2018; Partek Inc., St. Louis, MO, USA). Categorical variables were represented as *n*, and Chi-square test was used for inter-group comparisons in IBD patient cohorts. Statistical analyses were performed using one-way ANOVA and subsequent Tukey Post hoc test (*α* = 0.05, *p ≤ 0.05, **p ≤ 0.01, ***p ≤ 0.001).

## Supplementary information

Supplementary Tables, Supplementary Figure Legends

Supplementary Figure S1

Supplementary Figure S2

Supplementary Figure S3

Supplementary Figure S4

Supplementary Figure S5

## Data Availability

Transcript Profiling Sequencing data have been deposited at ArrayExpress (http://www.ebi.ac.uk/arrayexpress, accession numbers: E-MTAB-9405, E-MTAB-9409).
