# Supplementary material for: RNF20 and RNF40 regulate vitamin D receptor-dependent signaling in inflammatory bowel disease
Source: Cell Death Differ. 2021 Jun 4;28(11):3161–75. doi: 10.1038/s41418-021-00808-w (PMC8563960; doi:10.1038/s41418-021-00808-w)
Supplement: Supplementary file 1 — Supplementary Tables, Supplementary Figure Legends [file 41418_2021_808_MOESM1_ESM.docx]

**SUPPLEMENTAL TABLES**

**Supplementary Table S1: Characteristics of Crohn’s disease patients monitored for H2Bub1 levels using immunohistochemical staining.**

|  | **All**  (n=18) | **H2Bub1 loss** (n=14) | **H2Bub1 unchanged** (n=4) | **p-value** |
| --- | --- | --- | --- | --- |
| Female, n (%) | 6 (33.3%) | 4 (66.7%) | 2 (33.3%) | 0.4227 |
| BMI (± SD) | 24.2 (± 5.8) | 24.0 (± 4.9) | 25.1 (± 9.4) | 0.7480 |
| Smokers, n (%) | 2 (11.1%) | 2 (100%) | 0 (0.0%) | 0.4227 |
| Number of surgeries (± SD) | 1.8 (± 1.2) | 1.9 (± 1.4) | 1.5 (± 0.6) | 0.5609 |
| **Age (years)** | | | | |
| Diagnosis (± SD) | 32.1 (± 16.3) | 34.7 (± 17.7) | 22.8 (± 1.3) | 0.2039 |
| **Disease location** | | | | |
| Ileal, n (%) | 2 (11.1%) | 2 (100.0%) | 0 (0.0%) | 0.4227 |
| Colonic, n (%) | 2 (11.1%) | 1 (50.0%) | 1 (50.0%) | 0.3162 |
| Ileocolonic, n (%) | 14 (77.8%) | 11 (78.6%) | 3 (21.4%) | 0.8796 |
| **Disease behavior** | | | | |
| Inflammatory, n (%) | 18 (100%) | 14 (77.8%) | 4 (22.2%) | >0.9999 |
| Stenosing, n (%) | 10 (55.6%) | 7 (70.0%) | 3 (30.0%) | 0.3749 |
| Fistulizing, n (%) | 8 (44.4%) | 6 (75.0%) | 2 (25.0%) | 0.7998 |
| Tumor, n (%) | 0 (0.0%) | 0 (0.0%) | 0 (0.0%) | >0.9999 |
| **Therapy** | | | | |
| Immunosuppressants, n (%) | 9 (50%) | 7 (77.8%) | 2 (22.2%) | >0.9999 |
| TNF-α inhibitors, n (%) | 1 (5.6%) | 1 (100.0%) | 0 (0.0%) | 0.5823 |
| Steroids, n (%) | 4 (22.2%) | 3 (75.0%) | 1 (25.0%) | 0.8796 |
| NSAIDs, n (%) | 3 (16.7%) | 1 (33.3%) | 2 (66.7%) | 0.0425 |
| Others, n (%) | 1 (5.6%) | 1 (100.0%) | 0 (0.0%) | 0.5823 |

BMI: Body mass index, TNF-α: Tumor necrosis factor alpha, NSAIDs: Nonsteroidal anti-inflammatory drugs

**Supplementary Table S2: Characteristics of Crohn’s disease patients analyzed for H3K27ac occupancy.**

|  | **All**  (n=30) | **RNF20/40^low^** (n=16) | **RNF20/40^high^** (n=14) | **p-value** |
| --- | --- | --- | --- | --- |
| Female, n (%) | 17 (56.7%) | 9 (52.9%) | 8 (47.1%) | 0.9607 |
| BMI (± SD) | 24.4 (± 5.6) | 23.1 (± 3.4) | 28.6 (± 5.3) | 0.0019 |
| Smokers, n (%) | 14 (46.7%) | 5 (35.7%) | 9 (64.3%) | 0.0704 |
| Number of surgeries (± SD) | 0.7 (± 1.4) | 0.7 (± 1.3) | 0.8 (± 1.4) | 0.8407 |
| **Age (years)** | | | | |
| Diagnosis (± SD) | 27.7 (± 17.8) | 23.7 (± 16.9) | 32.4 (± 18.2) | 0.1855 |
| **Disease location** | | | | |
| Ileal, n (%) | 9 (30.0%) | 4 (13.3%) | 5 (16.7%) | 0.5229 |
| Colonic, n (%) | 0 (0.0%) | 0 (0.0%) | 0 (0.0%) | >0.9999 |
| Ileocolonic, n (%) | 21 (70.0%) | 12 (57.1%) | 9 (42.9%) | 0.5229 |
| **Disease behavior** | | | | |
| Inflammatory, n (%) | 30 (100.0%) | 16 (53.3%) | 14 (46.7%) | >0.9999 |
| Stenosing, n (%) | 27 (90.0%) | 14 (51.9%) | 13 (48.1%) | 0.6256 |
| Fistulizing, n (%) | 16 (53.3%) | 9 (56.2%) | 7 (43.8%) | 0.7321 |
| Tumor, n (%) | 0 (0.0%) | 0 (0.0%) | 0 (0.0%) | >0.9999 |
| **Therapy** | | | | |
| Immunosuppressants, n (%) | 4 (13.3%) | 2 (50.0%) | 2 (50.0%) | 0.8859 |
| TNF-α inhibitors, n (%) | 20 (66.7%) | 11 (55.0%) | 9 (45.0%) | 0.7958 |
| Steroids, n (%) | 21 (70.0%) | 9 (42.9%) | 12 (57.1%) | 0.0789 |
| NSAIDs, n (%) | 4 (13.3%) | 3 (75.0%) | 1 (25.0%) | 0.3508 |
| Others, n (%) | 5 (16.6%) | 3 (60.0%) | 2 (40.0%) | 0.7434 |

BMI: Body mass index, TNF-α: Tumor necrosis factor alpha, NSAIDs: Nonsteroidal anti-inflammatory drugs

**Supplementary Table S3: Mouse genotyping primers.**

| **Gene** | **Sequence (5'-3')** |
| --- | --- |
| *Rnf20* | TTCCCATGACATGCTAACGA |
|  | AGGCTTTTTCATCCACTGGT |
| *Rnf40* | GCGAAAGTCACATTTGGCCT |
|  | CTTCACATTCCGTTCCTGCC |
| Cre | CAAGCCTGGCTCGACGGCC |
|  | CGCGAACATCTTCAGGTTCT |

**Supplementary Table S4: Antibodies used for Western blot, ChIP and immunohistochemistry.**

| **Antibody** | **Host** | **Cat. no.** | **Company** |
| --- | --- | --- | --- |
| CD31 | Rat | DIA-310 | Diagenode |
| CD45 | Rat | 553089 | BD Biosciences |
| H2B | Rabbit | ab1790 | Abcam |
| H2Bub1 (IHC) | Mouse | Previously published^[22](#_ENREF_22)^ | |
| H2Bub1 (WB) | Rabbit | 55465 | Cell Signaling |
| H3K4me3 | Rabbit | C15410030 | Diagenode |
| H3K27ac | Rabbit | 8173 | Cell Signaling |
| HSC70 | Mouse | sc-7298 | Santa Cruz |
| MUC2 | Rabbit | sc-15334 | Santa Cruz |
| RNF20 | Rabbit | ab33500 | Abcam |
| RNF40 | Rabbit | ab191309 | Abcam |
| VDR | Rabbit | 12550 | Cell Signaling |

**Supplementary Table S5: qRT-PCR primers.**

| **Gene** | **Sequence (5'-3')** |
| --- | --- |
| *Rplp0* | GATCGGGTACCCAACTGTTG |
|  | CAGGGGCAGCAGCCGCAAA |
| *Abcb1a* | GCGACTCCGATACATGGTTT |
|  | ACCCTGTAGCCCCTTTCACT |
| *Galnt10* | TGACCGATGCCGAGAGAGT |
|  | AGAGAGCGATTCAGGGAGATT |
| *Galnt4* | CCACTCTGCTTCGGACCATT |
|  | GGACCAGCCCCTCTCTCTTA |
| *Muc2* | GCTGACGAGTGGTTGGTGAATG |
|  | GATGAGGTGGCAGACAGGAGAC |
| *Rnf20* | TGGCTAGCAGTTCCAGTGAAG |
|  | ACAATCTGGGACACAGCACG |
| *Rnf40* | TCCACGGAGGAGATGGATTC |
|  | CGATGAGGAGTGTGGCATCA |
| *Tspan3* | GCCAGCCGTGCTATTGACTA |
|  | CTGCCGTTACAGCTTTTGGC |
| *Vdr* | TACACCCCCTCACTGGACAT |
|  | ATCAGAGGTGAGGTCCCTGAA |
| *VDR* | CCTTCACCATGGACGACATG |
|  | CGGCTTTGGTCACGTCACT |

**Supplementary Table S6: ChIP-qPCR primers.**

| **Gene** | **Region** | **Sequence (5'-3')** |
| --- | --- | --- |
| *Abcb1a* | Summit | AGAGCCGCTTCTTCCAAAGTC |
|  |  | ACTCACGAGGACGCTGACA |
|  | Transcr. reg. | GGCTGTTGTCAATACCCCTGTT |
|  |  | ATGCAGATGCATATAGTCCAGGG |
|  | Negative | GCAGTTAGGAAGTGAGGGGC |
|  |  | AGGTCATTGGGGTCTGTCCT |
| *Galnt10* | Summit | CCGAGAGTTGGGAGTACTCTTTC |
|  |  | AGAGCAGACACGAAGGATAGGT |
|  | Transcr. reg. | CTAGCAGCTCTGGTGGAGGA |
|  |  | AGAAGGAAGGCTGGAGGTGT |
| *Tspan3* | Summit | CGCGCCGCACAGAGG |
|  |  | GCTGGTCTTCCTCAACCTCATC |
|  | Transcr. reg. | CCTGAGAACCTGAGGGTCAGT |
|  |  | ACACTACTGTTGGGTGTTTTCTGATA |
|  | Negative | AGAAGACAATGCTTGCCCATGT |
|  |  | GGTCTGACCCAACCATCGC |
| *Vdr* | Summit | AGCCTCGTACGAACTTCAGC |
|  |  | AAGTTGGCGACTGAGATCCG |
|  | Transcr. reg. | TAGGATGCAACACTCAACAGCA |
|  |  | GTAGAGGAAGGCCTAGGGTCTAA |
|  | Negative | TAGCTAGCAAAGACGCACATCC |
|  |  | ACTTCAGTGTAGCTCTTGCTTCAT |

**SUPPLEMENTAL LEGENDS**

**Supplementary Figure S1: Intestinal *Rnf20* and *Rnf40* knockout promotes inflammation.** (A) 14 days after the first tamoxifen injection, *Rnf20* and *Rnf40* knockout mice displayed severe weight loss and (B, C) increased disease activity. AUC: Area under the curve. (D) *Rnf20*^fl/fl^ and *Rnf40*^fl/fl^ mice were more likely to display diarrhea, but the (E) presence of occult blood was not affected. (F) Colon length was reduced in *Rnf20*^fl/fl^ and (G) *Rnf40*^fl/fl^ animals 14 days upon the first tamoxifen injection. (H) The knockout efficiency in *Rnf20*^fl/fl^ and *Rnf40*^fl/fl^ mice was demonstrated using IHC for RNF20 and RNF40 on colon sections. Scale bar: 100 µm. (I) H&E staining of colon sections revealed an increased presence of lymphoid aggregates in *Rnf20* knockout mice. (J, K) IHC for CD31 showed an inflammation-associated elevation in vascularization. Scale bar: 100 µm. (L) Only minor differences in serum IL-6 levels were detected among genotypes. One-way ANOVA, mean ± SEM.

**Supplementary Figure S2: Aging mice with a heterozygous *Rnf20* and *Rnf40* knockout do not develop spontaneous intestinal inflammation.** (A, B) The DAI of wild type as well as *Rnf20* and *Rnf40* heterozygous mice was determined weekly for 6 months and did not differ significantly among genotypes as evidenced by no effect on (C) weight loss, (D) stool consistency or (E) the presence of occult blood. (F) When sacrificing 6-month old animals, there was no difference in epithelial damage in the colon and, accordingly, (G) the H-score was similar among genotypes. The general absence of inflammation-associated signs indicated that heterozygous animals did not develop spontaneous colitis. (H) Representative images of H&E-stained colons of 6-month old mice. Scale bar: 100 µm. One-way ANOVA, mean ± SEM.

**Supplementary Figure S3: DSS-induced colitis is not exacerbated by heterozygous loss of *Rnf20* and *Rnf40* in the intestinal epithelium.** (A) Wild type and *Rnf20*/*40* heterozygous mice were treated with 0.75% DSS for 14 consecutive days and the DAI was similar among genotypes since no difference was detected in (B) weight loss, (C) stool consistency and (D) the presence of occult blood. (E, F) While colon length was slightly reduced in *Rnf40*^wt/fl^ mice, (G) no differences in epithelial damage and (H) H-score were observed between genotypes. (I) Representative images of H&E-stained colon sections after DSS treatment. Scale bar: 100 µm. (J) H2Bub1 staining intensity after IHC on colon sections was quantified using FIJI and no reduction was detected in heterozygous mice. One-way ANOVA, mean ± SEM.

**Supplementary Figure S4: The downregulation of VDR targets in *Rnf20* and *Rnf40* knockout IECs can be rescued by *VDR* overexpression.** (A) The mRNA expression of VDR-dependent genes was significantly reduced in *Rnf20*^fl/fl^ and *Rnf40*^fl/fl^ IECs (n=6) as detected using qRT-PCR. (B) IECs were transfected with a *VDR* expression or control plasmid and, after 48 hours, with siRNAs to deplete RNF20 and RNF40. After 72 hours, RNA was isolated and mRNA levels of *Vdr* and VDR target genes were analyzed using qRT-PCR (n=3). The downregulation of these genes upon RNF20 and RNF40 depletion was partially rescued after *VDR* overexpression.(C) Overexpression of human *VDR* was verified by qRT-PCR and subsequent agarose gel electrophoresis. One-way ANOVA, mean ± SEM.

**Supplementary Figure S5: RNF20/40 regulate VDR-associated gene expression in the colon but do not affect vitamin D serum levels.** (A) The downregulation of *Vdr* and VDR targets upon *Rnf20/40* deletion was confirmed in whole colon lysates (n=6) as detected using qRT-PCR. (B) Immunohistochemical staining for MUC2 and (C) analysis of staining intensity using FIJI revealed significantly reduced MUC2 levels in colon sections upon the knockout of *Rnf20* and *Rnf40*. Scale bar: 100 µm. (D) Analysis of vitamin D serum levels in Crohn’s disease patients with high or low expression of RNF20/40-dependent genes and in (E) our mouse model for spontaneous *Rnf20/40*-associated intestinal inflammation revealed no differences in vitamin D levels among cohorts. One-way ANOVA, mean ± SEM.
